# Body mass index and diet-related inflammation as predictors of sleep disorders: A cross-sectional study

**DOI:** 10.1097/MD.0000000000047924

**Published:** 2026-03-06

**Authors:** Yiren Bao, Bo Liang, Heran Zhou, Xueyan Huang, Yankai Dong, Rui Wang

**Affiliations:** a Department of Massage, The Hangzhou TCM Hospital Affiliated to Zhejiang Chinese Medical University, Hangzhou, Zhejiang, China; b Department of Nephrology, The Key Laboratory for the Prevention and Treatment of Chronic Kidney Disease of Chongqing, Chongqing Clinical Research Center of Kidney and Urology Diseases, Xinqiao Hospital, Army Medical University (Third Military Medical University), Chongqing, China; c Department of Oncology, The Hangzhou TCM Hospital Affiliated to Zhejiang Chinese Medical University, Hangzhou, Zhejiang, China; d Modern Industrial College of Traditional Chinese Medicine and Health, Lishui University, Lishui, Zhejiang, China.

**Keywords:** DII, machine learning, NHANES, nutrition, sleep disorders

## Abstract

This study examines diet as a key risk factor for sleep disorders and integrates physiological indicators to develop a machine learning (ML)-based model for targeted public health interventions. Data from 5158 2011 to 2014 National Health and Nutrition Examination Survey (NHANES) participants were analyzed. Dietary, lifestyle, and physiological variables used to build sleep disorder prediction models with random forest, extreme gradient boosting, light gradient boosting machine, and logistic regression. Model interpretability was assessed using Shapley additive explanations (SHAP). Key predictors were further analyzed using progressive modeling and least absolute shrinkage and selection operator (LASSO) regression. All ML models showed acceptable-to-excellent discrimination (area under the receiver operating characteristic curve: 0.744–1.000), with light gradient boosting machine achieving the highest performance (area under the receiver operating characteristic curve  = 1.000). SHAP analysis showed that dietary inflammatory index (DII), body mass index (BMI), and age were positively associated with sleep disorder risk, while mean arterial pressure was negatively associated. In progressively adjusted logistic regression models, BMI was consistently positively associated with sleep disorders (model 3 odds ratio [OR] = 1.065, 95% confidence interval [CI]: 1.050–1.080; *P* < .001), whereas DII was associated with sleep disorders primarily in less-adjusted models (model 1 OR = 1.099, 95% CI: 1.035–1.168; *P* = .002; model 2 OR = 1.072, 95% CI: 1.004–1.145; *P* = .037). To further identify which dietary components driving the DII-related signal were most relevant to sleep disorder risk, we applied LASSO to the nutrient components of DII, which selected iron, carbohydrates, and total fat as the major contributors to the diet-related sleep disorder risk profile. An interpretable ML model based on National Health and Nutrition Examination Survey data demonstrated good discrimination for sleep disorders and consistently highlighted BMI and DII as central correlates. SHAP and LASSO further translated these associations into clinically interpretable dietary signals, including iron, carbohydrate, and total fat intake within the DII framework, supporting screening-oriented risk profiling and prioritization of individuals for further sleep evaluation and targeted nutrition assessment.

## 1. Introduction

Quality sleep is crucial for both physical and mental health and plays a fundamental role in maintaining optimal brain function, while chronic sleep deprivation can result in significant cognitive and physiological impairments.^[1]^ Sleep disorders, affecting ~30% of adults worldwide, are characterized by persistent difficulties in obtaining sufficient sleep and are associated with numerous adverse health outcomes, including obesity, diabetes, hypertension, tachycardia, myocardial infarction, stroke, mood disorders, and fatigue.^[2-5]^ These disorders also impair decision-making abilities, reduce work efficiency and productivity, and increase the risk of accidents, occupational injuries, medical errors, and compromised healthcare quality.^[6]^ In the United States, the economic burden of sleep disorders is substantial, with estimated direct costs exceeding $160 billion annually and indirect costs surpassing $100 billion,^[2,3]^ highlighting their status as a major public health concern. The onset of sleep disorder is influenced by multiple risk factors, such as depression, female sex, advanced age, low socioeconomic status, marital status, ethnicity, and comorbid medical or psychiatric conditions.^[7]^ Although many studies have examined these factors, their relative importance and combined contributions remains unclear, warranting further investigation.

Regular circadian rhythm is essential for maintaining optimal sleep and is regulated by internal clock genes as well as external factors, particularly diet.^[8]^ Pro-inflammatory diets, characterized by high intake of refined sugars, trans-fats, and processed foods, can elevate inflammatory cytokines, disrupt sleep, and increase the risk of disorders such as insomnia and sleep apnea.^[9]^ The dietary inflammatory index (DII), which reflects the inflammatory potential of the diet, has been shown to be associated with poorer sleep outcomes, with higher DII scores linked to increased sleep disturbances.^[10]^ Conversely, the comprehensive dietary antioxidant index (CDAI), an indicator of dietary antioxidant capacity, has been linked to a lower incidence of sleep disorders, likely due to the neuroprotective and stress-reducing effects of antioxidants.^[11]^ Additionally, factors such as body mass index (BMI), age, physical activity and blood pressure also influence sleep by modulating metabolic pathways, neurotransmitter synthesis, vascular function and hormonal balance.^[2]^

Machine learning (ML), a powerful tool for data mining and predictive analytics, offers advantages over traditional statistical methods due to its capacity to model complex, non-linear relationships and interactions among variables.^[12,13]^ While prior research has typically focused on isolated factors, ML enables the integration and analysis of multidimensional data, facilitating the identification of latent risk factors and their interrelationships.^[14]^ Recent studies have applied ML to explore associations between environmental exposures (e.g., organophosphate ester metabolites) and sleep outcomes,^[15]^ as well as to predict obstructive sleep disorder risk based on demographic and clinical features.^[16]^ However, considerable gaps remain in the development of comprehensive predictive models for sleep disorders.

Therefore, this study used DII as a primary diet-related factor and integrated a comprehensive set of variables, including demographic characteristics, lifestyle factors and metabolic parameters, from the National Health and Nutrition Examination Survey (NHANES) database to develop and validate an interpretable ML-based risk prediction model for sleep disorders. This approach aims to enhance the identification of individuals with a higher likelihood of sleep disturbances and provide insights for dietary interventions aimed at improving sleep health.

## 2. Materials and methods

### 2.1. Data source and participants

**Figure 1.**
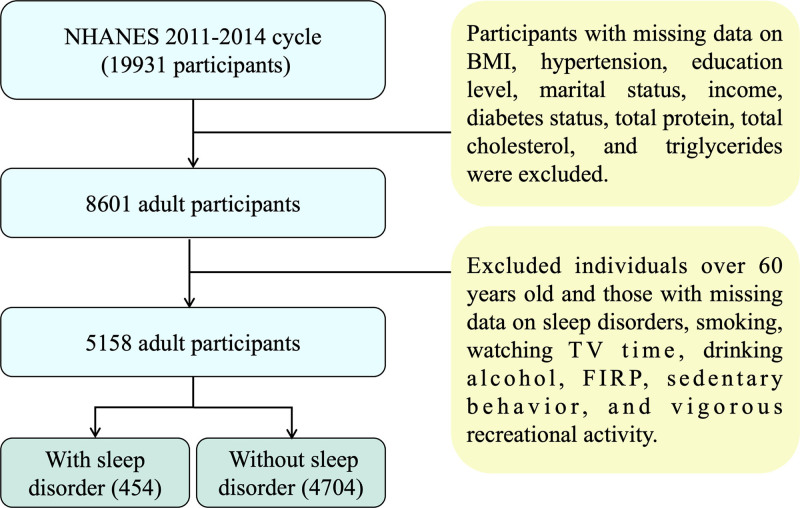
Flowchart of the sample selection from NHANES. BMI = body mass index, NHANES = National Health and Nutrition Examination Survey.

A cross-sectional study was conducted using data from the publicly available NHANES database. All participants provided informed consent either personally or through a proxy. We included 4 cognitive assessment tests to calculate composite cognitive function scores (*Z*-scores). This study initially included 19,931 participants from the 2011 to 2014 NHANES cycles, with demographic and clinical variables incorporated into the analysis. After excluding 11,330 individuals with missing data on key variables such as BMI, hypertension, diabetes, education level, marital status, household income, and laboratory indicators, an additional 3443 participants aged over 60 years were excluded due to missing information on smoking status, sleep disorders, physical activity, and computer usage. Ultimately, 5158 participants were included in the final analysis. Based on the presence or absence of self-reported sleep disorders, participants were categorized into 2 groups: a sleep disorder group (n = 454) and a non-sleep disorder group (n = 4704; Fig. 1).

### 2.2. Assessment of sleep disorder

Sleep disorders were identified from the medical records and self-reported diagnoses. Participants responding affirmatively to “Have you ever informed a physician or healthcare professional about having sleep disorders?” were classified as having sleep disorders in this study.^[17]^

### 2.3. Calculation of dietary inflammation index (DII)

This study analyzed 23 of the original 45 food parameters in the DII: alcohol, vitamin E, β-carotene, caffeine, carbohydrate, cholesterol, folic acid, dietary fiber, iron, energy, magnesium, monounsaturated fatty acids, niacin, protein, selenium, saturated fatty acids, total fat, vitamin A, vitamin B12, riboflavin, vitamin B6, vitamin C, and zinc. The DII calculation utilizes 24-hour dietary recall interviews or food records, with each food parameter referenced against standardized values in a global database.^[18]^ The 24-hour dietary recall data were multiplied by standard food parameters from the global database to calculate individual DII scores relative to the global mean. To minimize bias, these values were converted into percentiles, doubled, and reduced by one. Each food parameter’s percentile value was subsequently multiplied by its corresponding “overall food parameter-specific inflammatory effect score” to obtain individual food-specific DII scores. Finally, all individual food component DII scores were summed to derive each participant’s overall DII score.^[19]^

### 2.4. Comprehensive dietary antioxidant index (CDAI)

The development of the CDAI has been described previously^[20]^ and subsequently validated using serum anti-inflammatory markers in a separate prospective cohort study.^[21]^ For each participant, the CDAI was calculated by summing the intake of 6 antioxidants (*i*) derived exclusively from food sources (excluding dietary supplements). These antioxidants include vitamins A, C, and E, magnesium, zinc, and selenium, as follows:


$$\begin{array}{l} \;\;\;CDAI\;\;\;=\overset{6}{\underset{k=1}{\sum }}\frac{Xk\;\;\;-Uk}{Sk} \end{array}$$

where Xk represents the daily intake of antioxidant *k*; Uk denotes the mean value of Xk for antioxidant *k* across the entire cohort; and Sk is the standard deviation of Uk.

### 2.5. Metabolic dysfunction indicators

Blood pressure measurements were conducted at a mobile examination center. Fasting blood glucose, triglyceride, total cholesterol, and uric acid (UA) levels were measured using automated analyzers and enzymatic methods. Detailed measurement protocols are available on the NHANES website (https://www.cdc.gov/nchs/nhanes). Mean arterial pressure (MAP) was calculated as diastolic blood pressure + 1/3 × (systolic blood pressure − diastolic blood pressure).^[16]^ The triglyceride–glucose (TyG) index was calculated using a natural-log transformation as follows:


$$\begin{array}{l} TyG\;\;\;=\;\;\;ln\;\;\;\left(\frac{fasting\;\;\;triglycerides\;\;\;\left(mg/dL\right)\;\;\;\times \;\;\;fasting\;\;\;glucose\;\;\;\left(mg/dL\right)}{2}\right) \end{array}$$

We also constructed a metabolic score (MS) as the sum of the *z*-transformed values of 4 indicators: total cholesterol, UA, MAP, and TyG,^[22]^ where each *z*-score was computed as (value − sample mean)/sample standard deviation.

### 2.6. Covariates

Covariates were selected based on previous research and potential confounding. Specifically, we considered sociodemographic characteristics including age, sex, and race/ethnicity (Mexican American, other Hispanic, non-Hispanic White, non-Hispanic Black, and other Race including multi-racial), as well as socioeconomic and household indicators (ratio of family income to poverty, total number of people in the family, marital status, and annual family income).^[23]^ Educational level was operationalized as <9th grade, 9–11th grade, high school graduate/GED or equivalent, some college or AA degree, and college graduate or above.^[23]^ We also included health conditions (BMI, hypertension, and diabetes) and lifestyle factors (smoking status, drinking status, physical activity, sedentary behavior, and digital screen exposure based on available TV/video viewing and computer-use measures).^[23,24]^ In addition, UA and total protein were incorporated as biochemical covariates to reflect systemic metabolic and protein-metabolism status, given reported associations of sleep duration/quality with serum UA and of insomnia with circulating total protein in large population-based studies.^[23,25]^

### 2.7. Model development and validation

The SIMCA-P software was utilized to perform principal component analysis and orthogonal partial least squares discriminant analysis on the participants’ overall characteristics, aiming to differentiate between individuals with sleep disorders and healthy controls. Subsequently, ML models were employed to identify risk factors associated with sleep disorders through feature importance analysis. Four ML algorithms were implemented: extreme gradient boosting (XGBoost), random forest (RF), light gradient boosting machine (LightGBM), and linear regression. During model training, a 10-fold cross-validation approach was adopted, wherein the training dataset was partitioned into 10 subsets. For each iteration, 9 subsets were used for training, and the remaining subset served as the validation set. The final model accuracy was determined by averaging the accuracy scores from all 10 iterations. The model was evaluated using a test set comprising 30% of the original dataset. Model performance was assessed based on discriminative power and clinical utility, with the optimal model for each algorithm selected according to the highest area under the receiver operating characteristic curve. The discriminative performance of all 4 models was quantitatively evaluated using under the receiver operating characteristic metrics.

### 2.8. Shapley additive explanations (SHAP) visualization

Shapley additive explanations (SHAP) values were computed for the final selected ML model to quantify the contribution of each feature to predicted sleep disorder probability at both global and individual levels. Feature importance rankings and SHAP summary/dependence plots were used to visualize key predictors and their association patterns with the outcome.^[26]^

### 2.9. Statistical analysis

The data were categorized as continuous and categorical variables. Categorical variables were expressed as proportions (%), while continuous variables were described using either the mean (standard deviation) or median (interquartile range), as appropriate. To compare the differences between groups, 1-way ANOVA (for normally distributed data), Kruskal–Wallis test (for skewed distributions), and chi-square test (for categorical variables) were conducted. In our analytical approach, 3 progressive models were developed to examine the association between key predictors and sleep disorders. Model 1 with non-adjustment employed a generalized regression framework to estimate odds ratios with 95% confidence intervals (95% CIs). Model 2 incorporated adjustments for demographic and health-related covariates, including smoking, race/ethnicity, sex, educational, diabetes, and hypertension. Model 3 extended these adjustments to encompass socioeconomic indicators (marital status and annual family income), biochemical parameters (UA and total protein), and lifestyle factors (physical activity patterns, sedentary behavior, and digital screen exposure). All statistical analyses were performed using the R statistical computing platform (version 4.3.0) in conjunction with EmpowerStats analytical software, with statistical significance set at *P* < .05.

## 3. Results

### 3.1. Baseline characteristics of participants

**Figure 2.**
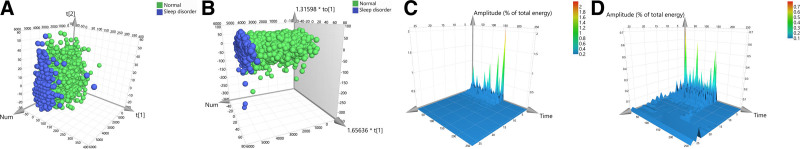
Multivariate analysis of comprehensive characteristics between sleep disorder patients and healthy participants. (A) Principal component analysis (PCA) score plot showing separation of participants by sleep disorder status. (B) Orthogonal partial least squares–discriminant analysis (OPLS–DA) score plot. (C) Wavelet power spectrum of PCA components. (D) Wavelet power spectrum of OPLS-DA components. Each point represents 1 participant; group colors correspond to sleep disorder status (as labeled in the figure). OPLS–DA = orthogonal partial least squares–discriminant analysis, PCA = principal component analysis.

A total of 5158 participants were included, comprising 454 with self-reported sleep disorders and 4704 without sleep disorder (Table 1; Fig. 1). Compared with controls (without sleep disorder), participants with sleep disorders were older (43.45 ± 11.06 vs 39.05 ± 11.89 years; *P* < .001) and had higher BMI (33.35 ± 8.92 vs 28.49 ± 6.79 kg/m^2^; *P* < .001). The sleep disorder group also had higher DII (0.723 ± 1.62 vs 0.48 ± 1.58; *P* = .003) and a lower ratio of family income to poverty (2.24 ± 1.66 vs 2.55 ± 1.69; *P* < .001). Lifestyle and comorbidity profiles differed, with higher current smoking (51.32% vs 40.20%; *P* < .001), greater sedentary time (427.05 ± 223.59 vs 391.45 ± 204.12 min/day; *P* = .003), and higher prevalence of hypertension (46.04% vs 22.28%; *P* < .001) and diabetes (18.06% vs 6.65%; *P* < .001) in the sleep-disorder group. Other baseline characteristics are shown in Table 1. Principal component analysis and orthogonal partial least squares discriminant analysis suggested separation of overall profiles between participants with and without sleep disorders (Fig. 2A–D).

**Table 1:** Baseline characteristics of study participants.

	Total (N = 5158)	With sleep disorder (n = 454)	Without sleep disorder (n = 4704)	*P*-value
Age	39.43 ± 11.88	43.45 ± 11.06	39.05 ± 11.89	<.001
Sex				
Male	2621 (50.81%)	228 (50.22%)	2393 (50.87%)	.791
Female	2537 (49.19%)	226 (49.78%)	2311 (49.13%)	
Race/ethnicity				
Mexican American	627 (12.16%)	39 (8.59%)	588 (12.50%)	.005
Other Hispanic	480 (9.31%)	47 (10.35%)	433 (9.21%)	
Non-Hispanic White	2105 (40.81%)	238 (52.42%)	1867 (39.69%)	
Non-Hispanic Black	1126 (21.83%)	97 (21.37%)	1029 (21.88%)	
Other race-including multi-racial	820 (15.90%)	33 (7.27%)	787 (16.73%)	
Education				
<9th grade	238 (4.61%)	20 (4.41%)	218 (4.63%)	.007
9–11th grade	636 (12.33%)	56 (12.34%)	580 (12.33%)	
High school graduate/GED or equivalent	1085 (21.04%)	113 (24.89%)	972 (20.66%)	
Some college or AA degree	1714 (33.23%)	170 (37.45%)	1544 (32.82%)	
College graduate or above	1485 (28.79%)	95 (20.93%)	1390 (29.55%)	
Ratio of family income to poverty	2.52 ± 1.69	2.24 ± 1.66	2.55 ± 1.69	<.001
Total number of people in the family				
1	1102 (21.37%)	115 (25.33%)	987 (20.98%)	.014
2	917 (17.78%)	84 (18.50%)	833 (17.71%)	
3	1002 (19.43%)	87 (19.16%)	915 (19.45%)	
4	954 (18.50%)	78 (17.18%)	876 (18.62%)	
5	645 (12.51%)	48 (10.57%)	597 (12.69%)	
6	286 (5.55%)	25 (5.51%)	261 (5.55%)	
7	252 (4.89%)	17 (3.74%)	235 (5.00%)	
Marital status				
Married	2475 (47.98%)	208 (45.82%)	2267 (48.19%)	.276
Widowed	67 (1.30%)	5 (1.10%)	62 (1.32%)	
Divorced	536 (10.39%)	86 (18.94%)	450 (9.57%)	
Separated	180 (3.49%)	23 (5.07%)	157 (3.34%)	
Never married	1399 (27.12%)	97 (21.37%)	1302 (27.68%)	
Living with partner	501 (9.17%)	35 (7.71%)	466 (9.91%)	
Annual family income				
$0–$4999	212 (4.11%)	17 (3.74%)	195 (4.15%)	<.001
$5000–$9999	271 (5.25%)	42 (9.25%)	229 (4.87%)	
$10,000–$14,999	378 (7.33%)	49 (10.79%)	329 (6.99%)	
$15,000–$19,999	342 (6.63%)	40 (8.81%)	302 (6.42%)	
$20,000–$24,999	422 (8.18%)	41 (9.03%)	381 (8.90%)	
$25,000–$34,999	596 (11.56%)	50 (11.01%)	546 (11.61%)	
$35,000–$44,999	516 (10.00%)	37 (8.15%)	479 (10.18%)	
$45,000–$54,999	393 (7.62%)	26 (5.73%)	367 (7.80%)	
$55,000–$64,999	289 (5.60%)	30 (6.61%)	259 (5.51%)	
$65,000–$74,999	254 (4.92%)	17 (3.74%)	237 (5.04%)	
$75,000–$99,999	479 (9.29%)	46 (10.13%)	433 (9.21%)	
$1,00,000 and over	1006 (19.50%)	59 (13.00%)	947 (20.13%)	
BMI	28.92 ± 7.13	33.35 ± 8.92	28.49 ± 6.79	<.001
Energy (kcal)	2265.28 ± 1048.66	2276.88 ± 1333.62	2264.16 ± 1017.11	.181
Total protein	71.56 ± 4.59	70.74 ± 4.68	71.64 ± 4.57	<.001
UA	5.37 ± 1.38	5.57 ± 1.54	5.35 ± 1.36	.003
MAP	87.39 ± 11.24	88.81 ± 11.25	87.25 ± 11.23	.005
DII	0.51 ± 1.59	0.723 ± 1.62	0.48 ± 1.58	.003
TyG	12.47 ± 1.07	12.72 ± 1.03	12.44 ± 1.07	<.001
MS	1.92 ± 0.75	2.08 ± 0.75	1.90 ± 0.75	<.001
CDAI	0.001 ± 4.28	−0.212 ± 4.561	0.021 ± 4.25	.106
Smoking				
No (never or past)	3034 (58.82%)	221 (48.68%)	2813 (59.80%)	<.001
Yes (current)	2124 (41.18%)	233 (51.32%)	1891 (40.20%)	
Drinking				
No (never or past)	1163 (22.55%)	106 (23.35%)	1057 (22.47%)	.669
Yes (current)	3995 (77.45%)	348 (76.65%)	3647 (77.53%)	
Hypertension				
No	3901 (75.63%)	245 (53.97%)	3656 (77.72%)	<.001
Yes	1257 (24.37%)	209 (46.04%)	1048 (22.28%)	
Diabetes				
No	4763 (92.34%)	372 (81.94%)	4391 (93.35%)	<.001
Yes	395 (7.66%)	82 (18.06%)	313 (6.65%)	
Minutes sedentary activity	394.58 ± 206.13	427.05 ± 223.59	391.45 ± 204.12	.003
Vigorous work activity				
No	4053 (78.58%)	350 (77.09%)	3703 (78.72%)	.42
Yes	1105 (21.42%)	104 (22.91%)	1001 (21.28%)	
Moderate work activity				
No	3228 (62.58%)	295 (64.98%)	2933 (62.35%)	.269
Yes	1930 (37.42%)	159 (35.02%)	1771 (37.65%)	
Vigorous recreational activity				
No	3587 (69.54%)	363 (79.96%)	3224 (68.54%)	<.001
Yes	1571 (30.46%)	91 (20.04%)	1480 (31.46%)	
Moderate recreational activity				
No	2805 (54.38%)	288 (63.44%)	2517 (53.51%)	<.001
Yes	2353 (45.62%)	166 (36.56%)	2187 (46.49%)	
Hours watch TV or video past 30 d				
0	704 (13.65%)	57 (12.56%)	647 (13.76%)	<.001
1	980 (19.00%)	62 (13.66%)	918 (19.52%)	
2	1351 (26.19%)	107 (23.57%)	1244 (26.45%)	
3	823 (15.96%)	62 (13.66%)	761 (16.18%)	
4	527 (10.22%)	54 (11.89%)	473 (10.06%)	
5	642 (12.45%)	94 (20.71%)	548 (11.65%)	
8	131 (2.54%)	18 (3.97%)	113 (2.40%)	
Hours use computer past 30 d				
0	1325 (25.69%)	128 (28.19%)	1197 (25.45%)	.833
1	1005 (19.48%)	75 (16.52%)	930 (19.77%)	
2	750 (14.54%)	61 (13.47%)	689 (14.65%)	
3	384 (7.45%)	24 (5.29%)	360 (7.65%)	
4	218 (4.23%)	21 (4.63%)	197 (4.19%)	
5	380 (7.37%)	43 (9.47%)	337 (7.16%)	
8	1096 (21.25%)	102 (22.47%)	994 (21.13%)	

Data are presented as mean ± SD for continuous variables and n (%) for categorical variables.

*P*-values were obtained using 2-sample *t*-tests for continuous variables and chi-square tests for categorical variables (2-sided).

Current smoking and current drinking were derived from NHANES questionnaire items and coded as yes (current)/no (never or past) based on participants’ current status at the time of survey (see Section 2 for detailed coding).

Vigorous/moderate work activity and vigorous/moderate recreational activity indicate self-reported engagement in the corresponding intensity activity (NHANES physical activity questionnaire).

BMI = body mass index, CDAI = continuous dietary antioxidant index, DII = dietary inflammatory index, MAP = mean arterial pressure, MS = metabolic score, NHANES = National Health and Nutrition Examination Survey, SD = standard deviation, TyG = triglyceride–glucose index, UA = uric acid.

### 3.2. Model evaluation and comparison

**Figure 3.**
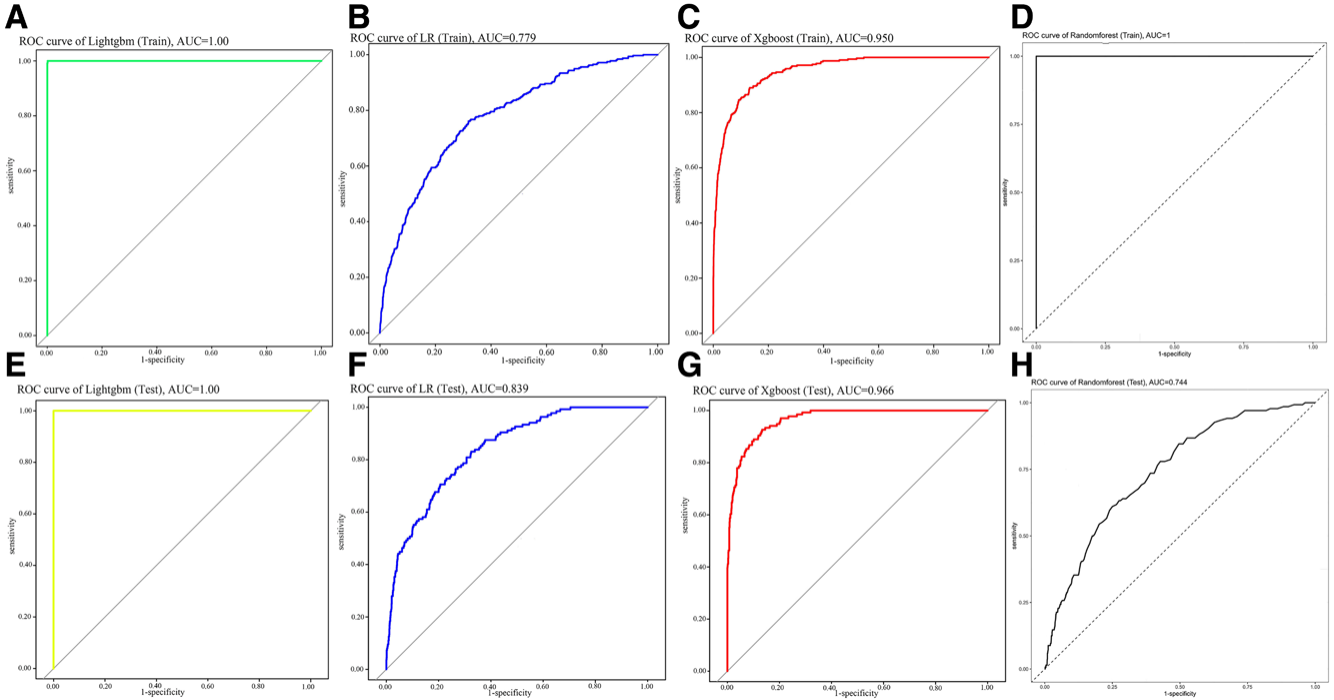
Receiver operating characteristic (ROC) curves of diverse machine learning algorithms. Training set ROC curves for (A) LightGBM, (B) logistic regression (LR), (C) XGBoost, and (D) random forest (RF). Testing set ROC curves for (E) LightGBM, (F) LR, (G) XGBoost, and (H) RF. Model discrimination is summarized by the area under the ROC curve (AUC; as shown in the figure). AUC = area under the curve, LightGBM = light gradient boosting machine, LR = logistic regression, RF = random forest, ROC = receiver operating characteristic.

**Figure 4.**
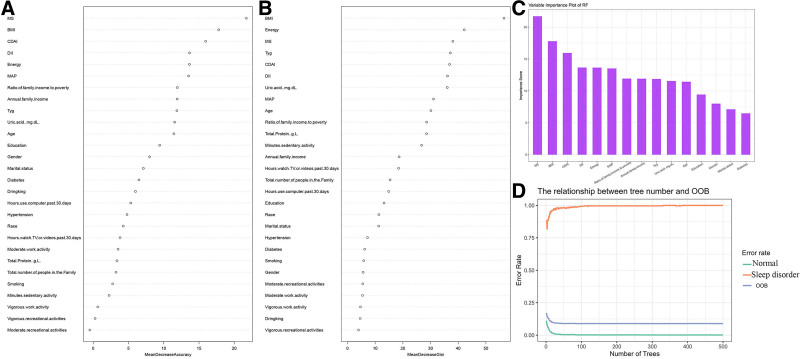
Random forest algorithm for significant feature identification: (A) Feature importance ranked by mean decrease in accuracy. (B) Feature importance ranked by mean decrease in Gini index. (C) Top 15 predictors ranked by importance. (D) Out-of-bag (OOB) error rate curve across trees, illustrating model stability. OOB = out-of-bag, RF = random forest.

Clinical and biochemical characteristics of the participants were utilized for model training and testing. All the models demonstrated AUC values ranging from 0.744 to 1 for the internal test set (Fig. 3). The LightGBM model exhibited the optimal performance (AUC [Train]: 1, AUC [Test]: 1), followed by XGBoost (AUC [Train]: 0.950, AUC [Test]: 0.966). Both RF (AUC [Train]: 1, AUC [Test]: 0.744) and logistic regression (LR; AUC [Train]: 0.779, AUC [Test]: 0.939) models demonstrated satisfactory performance. A feature-importance analysis based on the RF model is presented in Figure 4. The feature rankings according to the mean decrease accuracy and mean decrease Gini are shown in Figure 4A, B, respectively. The most significant features based on the mean decrease in accuracy were MS, BMI, CDAI, DII, and energy, whereas the mean decrease in Gini identified BMI, energy, MS, TyG, and CDAI as top predictors. A comprehensive ranking of the top 15 important features is shown in Figure 4C, with MS, BMI, CDAI, DII, and energy being the most prominent features. The error rate curve (Fig. 4D) demonstrated stable and accurate results for the 500 decision trees incorporated in this study.

### 3.3. Variable importance and variable interpretation

**Figure 5.**
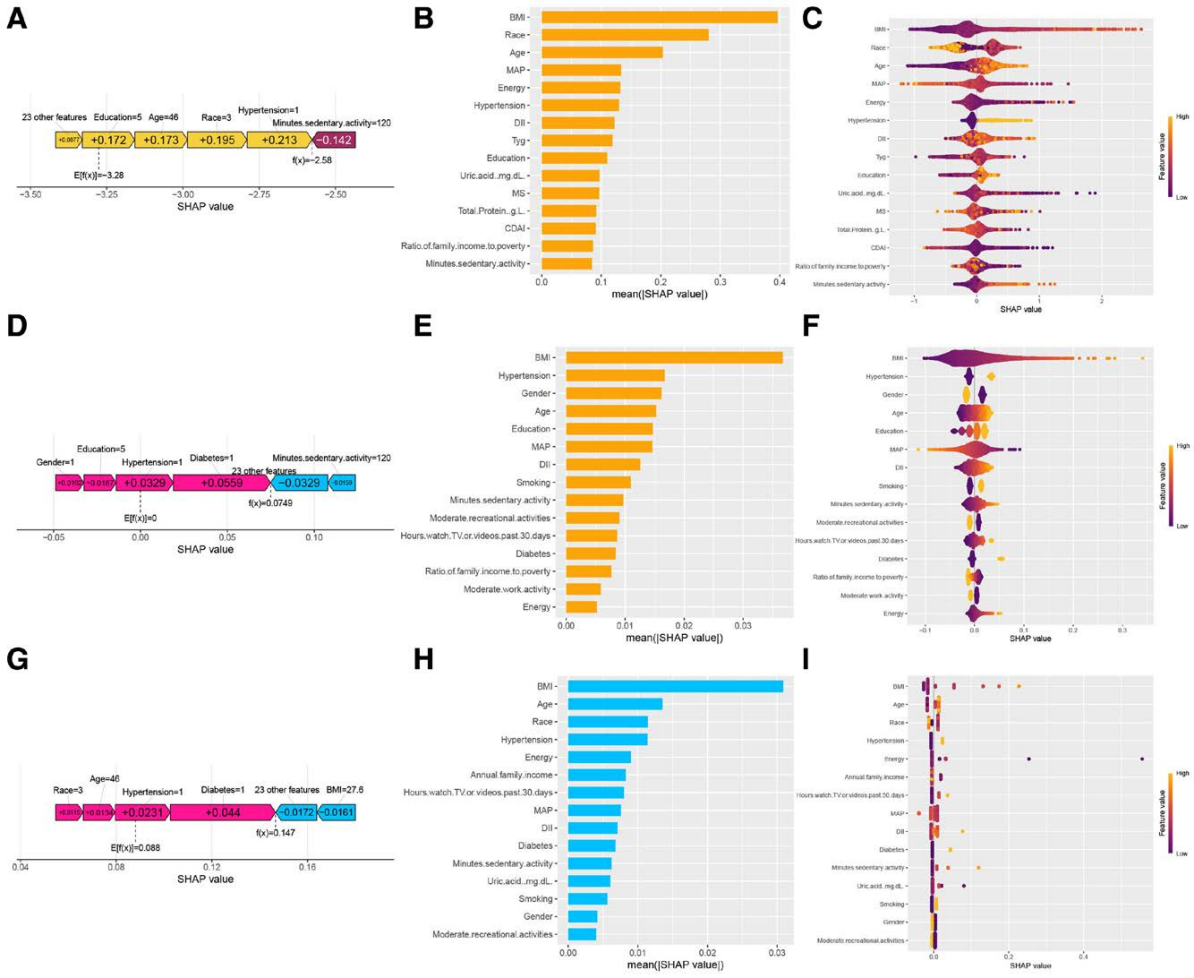
SHAP-based interpretation of model predictions across LightGBM, logistic regression, and XGBoost. (A) Example individual-level SHAP explanation for LightGBM (each feature’s contribution to the model output relative to the baseline). (B) Global feature importance from LightGBM based on mean absolute SHAP values. (C) SHAP summary (beeswarm) plot for LightGBM showing the direction and magnitude of feature contributions; each dot represents 1 participant, and color indicates the feature value (low to high). (D–F) Corresponding individual explanation, global importance, and beeswarm plots for logistic regression (LR). (G–I) Corresponding individual explanation, global importance, and beeswarm plots for XGBoost. Positive SHAP values indicate higher predicted sleep disorder risk, whereas negative values indicate lower predicted risk. LightGBM = light gradient boosting machine, LR = logistic regression, SHAP = Shapley additive explanations.

**Figure 6.**
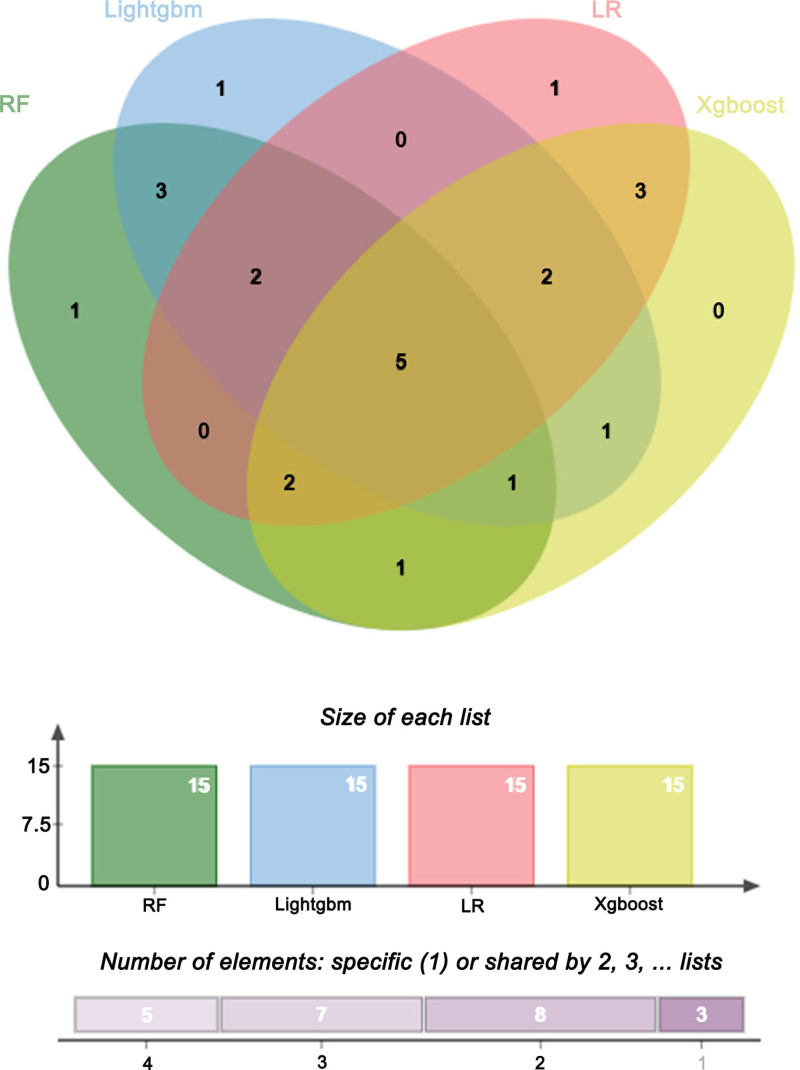
Overlap of key predictors across 4 machine learning models. Venn diagram illustrating overlapping top-ranked predictors identified by LightGBM, logistic regression (LR), random forest (RF), and XGBoost. Five features were shared across all 4 models: DII, BMI, CDAI, MS, and energy intake. BMI = body mass index, CDAI = continuous dietary antioxidant index, DII = dietary inflammatory index, LR = logistic regression, MS = metabolic score, RF = random forest.

A randomly selected sample was subjected to SHAP analysis to examine instance-level influencing factors and identify individual heterogeneity. The vertical axis represents the feature values, and the horizontal axis indicates the SHAP values, where *E*[*f*(*x*)] denotes the expected prediction value across all samples. Figure 5A illustrates the LightGBM model results, where yellow and red regions indicate positive and negative influences on predicted sleep disorders, respectively. A sample with *f*(*x*) = −2.58, exceeding *E*[(*f*(*x*)], suggests above-average sleep disorder severity. The SHAP interpretation identified education, age, race/ethnicity, hypertension, and sedentary activity as the primary contributing factors. Figure 5B shows the top 15 features selected by LightGBM, with BMI, race/ethnicity, age, MAP, and energy being the most significant. The SHAP beeswarm plot provides both feature importance rankings and their directional influence on model predictions.^[27]^ In the SHAP beeswarm plot, each row represents a variable, with the x-axis indicating SHAP values. The features are ranked by SHAP value importance from top to bottom. Each dot corresponds to an individual sample, where yellow indicates higher feature values and purple represents lower values. Positive SHAP values positively contributed to model predictions, whereas negative values exerted a negative influence. As shown in Figure 5C, BMI emerged as the most significant factor, demonstrating a positive correlation between a higher BMI and an increased risk of sleep disorders. In the LR model, pink regions indicate a positive influence on predicted sleep disorders, whereas blue regions represent negative influences (Fig. 5D). A sample with *f*(*x*) = 0.0749, which exceeds *E*[*f*(*x*)], suggests an above-average sleep disorder severity. SHAP analysis identified sex, education, hypertension, diabetes, and sedentary activity as the primary contributing factors. The top 15 features of LR are shown in Figure 5E, with BMI, hypertension, sex, age, and educational level being the most significant. The beeswarm plot of the LR model (Fig. 5F) highlighted BMI as the most influential factor, demonstrating a positive correlation between BMI and sleep disorder risk. Similarly, in the XGBoost model (Fig. 5G), the pink and blue regions represent the positive and negative influences, respectively. A sample with *f*(*x*) = 0.147, above *E*[*f*(*x*)], indicates elevated sleep disorder severity. The SHAP interpretation identified age, race/ethnicity, hypertension, diabetes, and BMI as key determinants. Figure 5H shows the top 15 XGBoost features, with BMI, age, race/ethnicity, hypertension, and energy being the most prominent. The XGBoost beeswarm plot (Fig. 5I) confirmed BMI as the primary risk factor, showing an increased sleep disorder risk with higher BMI values. Notably, the SHAP case analysis revealed model-specific variations in the key influencing factors, demonstrating different feature selection patterns across the models. We identified 5 common features shared by all 4 models using Venn diagram analysis (Fig. 6): DII, BMI, CDAI, MS, and energy.

### 3.4. Interaction analysis of key features

**Figure 7.**
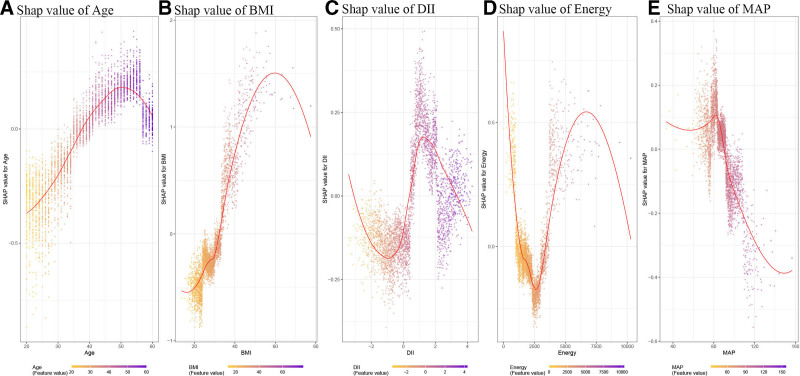
SHAP dependence plots for key predictors. (A) Age, (B) BMI, (C) DII, (D) energy intake, and (E) MAP. SHAP dependence plots showing the relationship between predictor values (*x*-axis) and SHAP values (*y*-axis) for selected key features. Each dot represents 1 participant; patterns indicate how the feature value relates to the predicted sleep disorder risk within the model. BMI = body mass index, DII = dietary inflammatory index, MAP = mean arterial pressure, SHAP = Shapley additive explanations.

**Figure 8.**
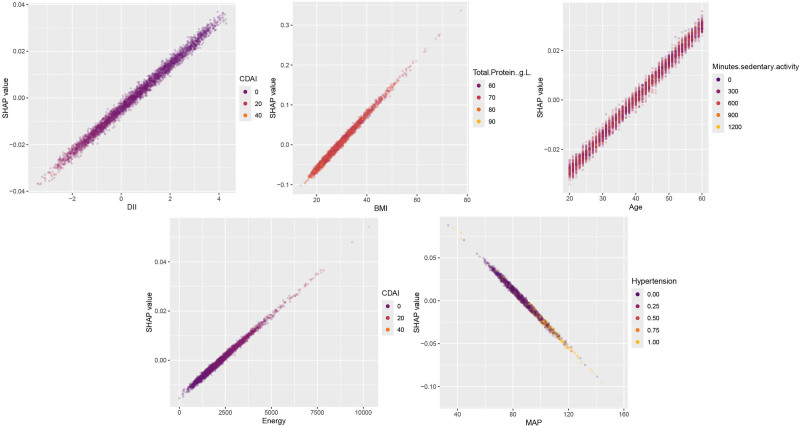
Visualization of interaction effects among important features. Visualization of pairwise interaction effects among important predictors using SHAP interaction values, highlighting whether the contribution of 1 feature to predicted sleep disorder risk varies across levels of another feature. SHAP = Shapley additive explanations.

Fig 7 illustrates the variation in the SHAP values across different feature levels for the key predictors. The analysis revealed a positive correlation between increasing BMI and age with an elevated risk of sleep disturbances, whereas MAP demonstrated a similar risk escalation pattern. The DII and energy intake exhibited fluctuating SHAP values across their respective ranges. Furthermore, the SHAP interaction value analysis was employed to examine the interplay among BMI, age, DII, energy intake, and MAP levels. As depicted in Figure 8, hypertension, CDAI, and sedentary behavior were identified as risk factors for sleep disorders, whereas total protein intake demonstrated protective effects. Notably, higher MAP levels were associated with increased SHAP interaction values in normotensive individuals. Importantly, elevated total protein consumption was found to mitigate the sleep-disruptive effects associated with higher BMI.

### 3.5. Association between important features and sleep disorder

**Figure 9.**
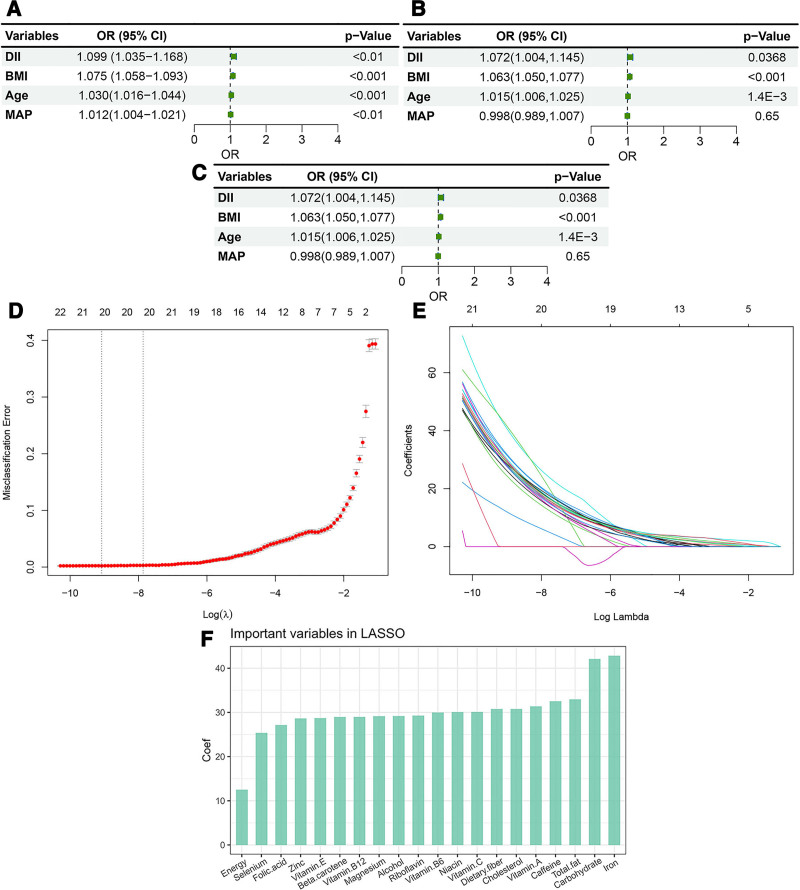
Regression-based associations of key predictors with sleep disorders and LASSO selection of DII-related nutrient components. (A–C) Associations between key predictors and sleep disorders from progressively adjusted logistic regression models (models 1–3; definitions provided in Section 2), presented as odds ratios with 95% confidence intervals. (D–E) LASSO regression for selecting nutrient components contributing to the DII-related dietary signal relevant to sleep disorder status (e.g., coefficient path and/or cross-validation). (F) Regression coefficients for nutrients selected by LASSO. DII = dietary inflammatory index, LASSO = least absolute shrinkage and selection operator.

Based on the significant features derived from ML and univariate analysis (*P* < .05), we selected BMI, age, DII, and MAP for correlation analysis with sleep disorders. Univariate generalized linear regression revealed significant associations between sleep disorders and DII, age, BMI, and MAP (all *P* < .001; Fig. 9A). After adjusting for smoking, race/ethnicity, sex, education, diabetes, and hypertension, MAP and age lost its significance, whereas DII (*P* = .037), and BMI (*P* < .001) remained significantly associated (Fig. 9B). Further adjustments for additional covariates (hypertension, education, diabetes, marital status, annual family income, ratio of family income to poverty, total protein, UA, sedentary activity, various physical activity levels, screen time, sex, race/ethnicity, and smoking status) revealed that only BMI was consistently significantly associated with sleep disorders (*P* < .001; Fig. 9C). Subsequently, the DII was dichotomized at a cutoff of 0, where 0 indicated a dietary intake equivalent to the global average. Least absolute shrinkage and selection operator (LASSO) regression ranked the importance of nutritional elements contributing to the DII, with iron emerging as the most significant contributor, followed by carbohydrates and total fat (Fig. 9D, F).

## 4. Discussion

Using NHANES 2011–2014 data, we integrated demographic, socioeconomic, lifestyle, dietary, and cardiometabolic information from 5158 adults to develop and compare 4 ML models for predicting self-reported sleep disorders. All models showed good discriminative performance, with LightGBM and XGBoost achieving the highest AUCs in the internal split (test-set AUC = 1.000 and 0.966, respectively). To enhance interpretability, SHAP was used to quantify feature contributions and identify a compact set of consistently influential predictors across models, led by BMI and diet-related inflammation features (DII-related signals) alongside key diet/metabolic variables. In complementary regression analyses, BMI remained robustly associated with sleep disorders after adjustment, whereas DII showed associations mainly in less-adjusted models, and nutrient-component LASSO further highlighted iron, carbohydrates, and total fat as prominent contributors to the DII-related dietary signature.

Previous research indicated that the factors associated with sleep disorders remain controversial.^[28]^ ML, a sophisticated branch of artificial intelligence, employs complex mathematical algorithms to analyze and classify patterns within diverse datasets, thereby supporting decision-making processes. Despite the efficacy of ML algorithms, their opaque reasoning mechanisms and inherent complexity in interpretability pose significant challenges to their practical application in medical decision-making.^[29]^Based on 4 ML models, this study identified 5 important features predictive of sleep disorders. Among these, 4 features demonstrated a significant influence: DII, BMI, age, and MAP.

BMI showed the most robust and clinically interpretable association with sleep disorders in our study. Across ML explainability analyses, SHAP consistently ranked BMI as the leading contributor, and BMI remained associated with sleep disorders after multivariable adjustment, supporting a dose–response pattern whereby higher BMI was linked to a greater likelihood of sleep disorders. This finding is consistent with prior population-based evidence that adiposity is related to sleep disturbances. Although adjusted odds ratios (ORs) may appear numerically close to 1 when expressed per 1-unit change, this scale can obscure clinical meaning. For example, an adjusted OR of 1.065/1 kg/m^2^ implies that a 5 kg/m^2^ higher BMI corresponds to ~1.37-fold higher odds, and a 10 kg/m^2^ difference corresponds to ~1.88-fold higher odds. Therefore, while the per-unit effect is modest, BMI differences within plausible clinical ranges can translate into materially different risk. Importantly, BMI is an inexpensive and routinely available measure, making it a practical marker for opportunistic risk stratification in clinical and public health settings; individuals with higher BMI may warrant more proactive assessment for sleep-related symptoms and comorbid sleep-disordered breathing.^[30]^

Diet-related inflammation, captured by the DII, was repeatedly prioritized by ML interpretation; however, its independent association with sleep disorders was modest and attenuated after full covariate adjustment. This pattern suggests that the observed DII and sleep relationship may partly reflect shared correlations with adiposity, cardiometabolic status, and socioeconomic or lifestyle factors, rather than a stable independent effect. DII was originally developed as a literature-derived measure of the inflammatory potential of diet, linking dietary exposures to circulating inflammatory biomarkers and related health outcomes.^[31]^ Consistent with prior population-based evidence, higher DII has been associated with non-recommended sleep duration and self-reported sleep disturbance.^[32]^ Evidence from other adult cohorts likewise suggests that more pro-inflammatorydiets may relate to poorer sleep quality (e.g., the MEAL study in Southern Italian adults).^[33]^ Mechanistically, inflammatory and psychosocial pathways may both be relevant; notably, Ren et al found that depressive symptoms mediated 24.06% of the association between empirical DII and sleep patterns.^[34]^ Finally, given that effect sizes were small when expressed per 1-unit increase in DII (ORs close to 1), any independent DII contribution likely has limited clinical impact at the individual level when interpreted on a per-unit scale, and may be more appropriately viewed as part of a broader metabolic with inflammatory risk profile.

To improve interpretability at the nutrient level, we applied LASSO to the nutrient components underlying the DII-related dietary signal and identified iron, carbohydrates, and total fat as the most prominent contributors. Iron is an essential trace element in various cellular proteins and biological processes. As a prosthetic component (e.g., for heme and iron-sulfur clusters), it plays a crucial role in oxygen storage and transport, electron transport in the respiratory chain, the Krebs cycle, regulation of gene expression, lipid metabolism, and DNA synthesis and replication.^[35]^ Iron deficiency is also associated with sleep disorders. Leung et al investigated the relationship between iron deficiency and the type and severity of sleep disorders, as well as whether iron supplementation could improve sleep-related symptoms. Their research suggested that iron supplementation was beneficial for the treatment of general sleep disorders.^[36]^ Iron is involved in cellular metabolism, DNA synthesis and repair, cell growth, and death.^[4]^ Despite its importance in life processes, excess iron is highly toxic as it activates the Fenton reaction, in which iron reacts with hydrogen peroxide to produce hydroxyl radicals. These toxic radicals damage cellular proteins, lipids, and nucleic acids.^[4,37]^ In addition, iron homeostasis regulates the immune system by modulating the activity of innate and adaptive immune cells, including T and B cells, neutrophils, and macrophages. Therefore, iron metabolism is tightly regulated, and its dysregulation has been implicated in the onset of various diseases. Iron metabolism is tightly regulated at the cellular level to maintain physiological intracellular and circulating iron concentrations. Dysregulation of iron metabolism can lead to increased intracellular iron concentrations, which are closely linked to inflammatory processes.^[38]^ Considering that inflammation plays an important role in the pathogenesis of sleep disorders,^[39]^ The results of our research indicate that the positive correlation between iron and DII suggests daily dietary iron intake should be carefully monitored to avoid excessive consumption.

Carbohydrate intake also exerts a notable influence on sleep physiology. Studies have shown that slow-wave sleep and rapid eye movement sleep, the 2 distinct stages of sleep, are inversely related to the consumption of fat and carbohydrates. Increased carbohydrate intake has been found to negatively affected rapid eye movement sleep and slow-wave sleep.^[40]^ Reducing carbohydrate intake, particularly before bedtime, decreases the secretion of inflammatory markers, which promotes clock gene expression and improves sleep regulation.^[41]^ Low-carbohydrate diet was significantly associated with improved sleep quality. Consuming high-carbohydrate foods, such as sweet, noodles, rice, and sugar-sweetened beverages is linked to poor sleep quality. A notable correlation was observed between reduced sleep quality and increased carbohydrate intake. Short sleep duration may contribute to insulin resistance, a key factor in the pathophysiology of metabolic syndrome.^[40]^

Total fat intake, particularly from saturated fats, is similarly implicated in poor sleep quality. Higher fat consumption has been associated with reduced sleep efficiency and increased sleep latency. Adolescents with high-fat diets or night-eating syndrome tend to report lower sleep quality and consume greater amounts of dietary fat.^[42]^ Saturated fat intake, in particular, has been linked to an increased risk of insomnia and daytime sleepiness. Beyond its direct effects on sleep, excessive fat intake contributes to obesity, which is a well-established risk factor for sleep disorders, including obstructive sleep apnea.^[43]^

With respect to other predictors, age was repeatedly highlighted in model interpretation, consistent with epidemiologic evidence that sleep disorders become more prevalent with increasing age.^[34]^ As with BMI, effect sizes are more interpretable when presented over meaningful increments (e.g., per 10 years rather than per year). MAP appeared influential in some ML models and interaction analyses, but it was not consistently associated after multivariable adjustment, suggesting that its importance may reflect interactions or correlations with broader cardiometabolic profiles (e.g., hypertension, diabetes, sedentary behavior) rather than a stable independent association. This model-dependent behavior reinforces the need to distinguish predictors that are consistently robust across analytic frameworks from those that may capture correlated risk clusters.

This study has several limitations. First, sleep disorders were assessed via self-reported questionnaire responses rather than clinical diagnoses, and we could not subtype disorders or quantify severity, which may introduce misclassification. Second, dietary intake was based on 24-hour recall, which may not reflect habitual diet and is subject to recall bias. Third, exclusion of participants with missing data may introduce selection bias. Fourth, the very high AUC observed for LightGBM (including AUC = 1.000 in the test set) raises the possibility of overfitting; therefore, external validation is needed to confirm generalizability.

## 5. Conclusion

Our study effectively employed ML strategies to explore the associations between dietary patterns, physiological factors, socioeconomic status, and sleep disorders among NHANES participants from 2011 to 2014. Our findings highlight the combined influence of DII and BMI in predicting sleep disorders. Additionally, LASSO regression on the nutritional components of DII emphasized the importance of adequate intake of iron, carbohydrates, and total fat in improving sleep disorders.

## Acknowledgments

We would like to thank NHANES for providing publicly available data, and most importantly. We also appreciate Yuhai Shou (graduated from the University of Pennsylvania) for his expert assistance in the writing of article.

## Author contributions

**Conceptualization:** Yankai Dong, Rui Wang.

**Data curation:** Heran Zhou.

**Funding acquisition:** Rui Wang.

**Formal analysis:** Yiren Bao, Bo Liang.

**Methodology:** Yiren Bao, Yankai Dong.

**Project administration:** Xueyan Huang.

**Writing – original draft:** Yiren Bao.

**Writing – review & editing:** Bo Liang, Rui Wang.
